# Deep Learning for Smart Healthcare—A Survey on Brain Tumor Detection from Medical Imaging

**DOI:** 10.3390/s22051960

**Published:** 2022-03-02

**Authors:** Mahsa Arabahmadi, Reza Farahbakhsh, Javad Rezazadeh

**Affiliations:** 1 North Tehran Branch, Azad University, Tehran 1667914161, Iran; arabahmadimahsa@gmail.com; 2 Institut Polytechnique de Paris, Telecom SudParis, 91000 Evry, France; reza.farahbakhsh@it-sudparis.eu; 3 Kent Institute Australia, Sydney, NSW 2000, Australia

**Keywords:** smart healthcare, brain tumor classification, MRI, deep neural networks, CNN, GAN, transfer learning

## Abstract

Advances in technology have been able to affect all aspects of human life. For example, the use of technology in medicine has made significant contributions to human society. In this article, we focus on technology assistance for one of the most common and deadly diseases to exist, which is brain tumors. Every year, many people die due to brain tumors; based on “braintumor” website estimation in the U.S., about 700,000 people have primary brain tumors, and about 85,000 people are added to this estimation every year. To solve this problem, artificial intelligence has come to the aid of medicine and humans. Magnetic resonance imaging (MRI) is the most common method to diagnose brain tumors. Additionally, MRI is commonly used in medical imaging and image processing to diagnose dissimilarity in different parts of the body. In this study, we conducted a comprehensive review on the existing efforts for applying different types of deep learning methods on the MRI data and determined the existing challenges in the domain followed by potential future directions. One of the branches of deep learning that has been very successful in processing medical images is CNN. Therefore, in this survey, various architectures of CNN were reviewed with a focus on the processing of medical images, especially brain MRI images.

## 1. Introduction

In this survey, we review studies on analyzing medical imaging with a focus on deep learning models.

**Figure 1:**
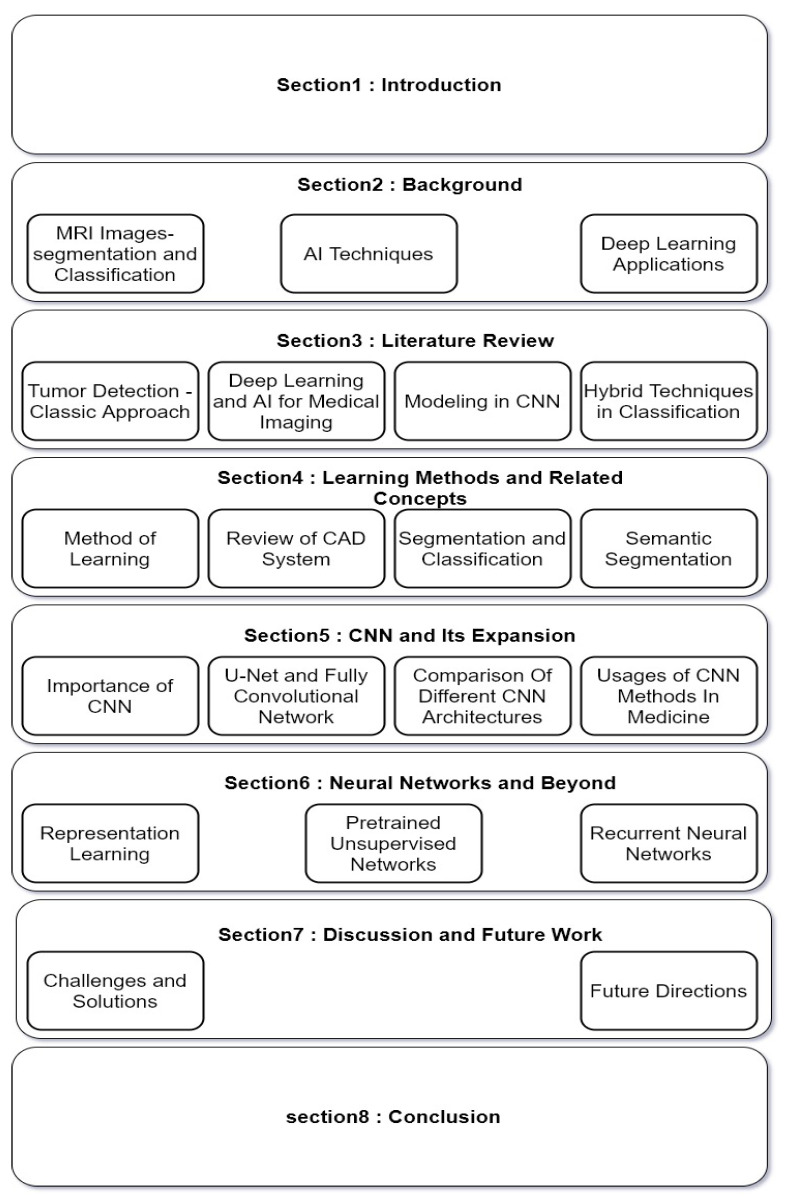
The structure of this survey.

Figure 1 illustrates the structure of this survey. We start by reviewing the subjects related to the topic of this study in Section 2. Next, we study the existing surveys on CNN from different aspects in Section 3. Section 4 discusses learning methods and CAD systems. To understand better the details on deep learning applications in the subject of this study, in Section 5, the importance and benefits of CNN-based studies are investigated, and it is completed by Section 6, where different neural networks methods are studied. Lastly, in Section 7, existing challenges in the topic of this survey and some solutions are discussed, which lead us to some future direction.

In this introduction section, we cover the following items: (1) the prevalence of brain tumors and their importance and impact on people’s lives; (2) overview the existing methods for diagnosing brain tumors and the importance of using MRI; (3) identify the problems that exist in the diagnosis of traditional methods and the importance of using technology in diagnosis; and (4) provide different deep learning methods used in MRI images and the importance of using artificial intelligence in image processing.

Brain tumors can hugely impact the quality of a patient’s lifetime and affect the whole of life because they have lasting and life-altering physical and psychological impacts. The recent development in smart healthcare and the application of artificial intelligence (AI) in radiology have shown remarkable progress in image-recognition tasks to identify different objects in imaging data automatically. Additionally, the recent development in smart healthcare and the application of artificial intelligence (AI) has been notable in image-recognition tasks in radiology. AI methods are good at automatically recognizing complex patterns in imaging data. Numerous applications which move the field forward at a fast speed have been found from a range of methods. The most common method to identify brain tumors is based on magnetic resonance imaging (MRI). MRI is used in medical imaging to display abnormal tissues in the body. Different techniques and methods are developed for brain tumor image segmentation and classification, but still this topic is a hot research line because the important issue is accuracy. Each year, we witness new methods for image segmentation that can handle limitations in the previous methods. The classification and segmentation based on deep learning are considered the best methods for identifying and extracting MRI image features. Artificial neural networks (ANNs) are inspired by the brain and neurons; artificial neurons are connected and can perform calculations on their certain inputs. If the output layer produces the final classification category, it means that ANN is used as a classifier. The convolutional neural network (CNN) is a type of deep neural network. In image segmentation and classification tasks, CNNs’ efficiency is great because they can extract features from the images.

The CBTRUS (Central Brain Tumor Registry of the United States) reported in 2012 that brain tumors in teenagers under 20 is the second leading cause of cancer-related deaths and in adults between 20 and 39 years of age are the fifth cause of cancer-related deaths. Based on braintumor.org estimation(https://braintumor.org/brain-tumor-information/brain-tumor-facts/ (accessed on 16 January 2022)) in 2022, 88,970 people will take an early brain tumor diagnosis. “braintumor.org” (accessed on 16 January 2022). In addition, an estimated 59,040 will be non-malignant (benign) and 25,130 will be malignant. The average age of diagnosis of a primary brain tumor is 60 years. Based on https://www.cancer.net/cancer-types/brain-tumor/statistics report (accessed on 16 January 2022), about 3540 children under the age of 15 will also be diagnosed with a brain or CNS tumor in 2020. A brain tumor is a big treatment for humans. A tumor is a growth of abnormal cells in the brain. The tumors are divided into two types. Benign, which are non-cancerous, and malignant, which are cancerous tumors. Unfortunately, the main concern is malignant brain tumors, as their growth is fast, they metastasize to other areas of the brain and spine, and are life threatening. The early detection of a tumor is a significant help in its treatment; different methods are available to diagnose this disease, but using different methods of imaging is the first way that doctors use them because they can find the tumor immediately.

Many common methods, such as X-ray, ultrasonography, computed tomography (CT) and magnetic resonance imaging (MRI), are used for imaging in medical imaging, but they cannot present the detailed and complete aspects and areas of brain tumors; however, they also improve the estimation of doctors about tumor treatment and growth [1].

Still, MRI is one of the most commonly used diagnostic methods in brain tumors in the clinical community because it is efficient in detecting brain tumors because of its high resolution, and it produces no harmful radiation and is a non-invasive method. It can also detect nervous disease. However, patients, when using MRIs, already feel that pain and/or several symptoms have appeared. With series of MRI images at different levels, doctors can determine the progress of the disease. The sequence of images are helpful, but we are looking for accurate and sensitive methods to find a way for quick and accurate detection. Normal brains are made of three types of tissues: white matter, gray matter, and the cerebrospinal fluid. T1 and T2 are the most usual MRI sequences, and each provides particular details and information about tissues. T1-weighted MRI (T1) is related to contrast enhancement and T2-weighted MRI (T2) is related to fluid-attenuated inversion recovery [2]. MRI can show different areas of the brain, so used in brain tumor segmentation studies, it contains image textures, local histograms, and structure tensor eigenvalues. MRIs can be provided in manual, semi-automatic, and fully automatic techniques, and are related to human interactivity [3].

There are known challenges with the traditional and manual methods of tumor diagnosis, as follows. (1) Diagnosis is generally based on the physician’s experience and needs complete user supervision. (2) Normally, it is accurate when the sickness is advanced, but not as accurate as semi-automatic methods in the very early stages of the problem. (3) Finally, it is also a time-consuming process, motivating the search for a better solution. In some cases, the border of the tumor is not recognized correctly, so the tumor is not completely emptied and causes the tumor to grow again. The manual process has the same challenge of being time consuming, so semi-automatic methods are acceptable by radiologists in the clinic.

In this study, we overview the main efforts on applying different types of deep learning methods on the MRI available data and provide the existing challenges in the domains with some potential future directions. Table 1 presents the most cited surveys in the domain related to our study. It also describes the distinguishing features of each survey to our study. Based on our knowledge and considering the previous efforts, especially the available surveys in the domain, we believe that our study adds valuable insight to this research direction.

The use of artificial intelligence in radiology reduces error rates more than human works [10]. With developments in recurrent, feed-forward, and convolutional neural networks (CNNs), artificial neural networks fields have become very popular because in pattern recognition contests, they achieve good results, and advanced versions of ANNs are implemented commonly on graphics processing units.

A variety of image-processing techniques and methods have been used to diagnose abnormalities in the brain. For example, SVM (support vector machine) is used for the automatic segmentation of brain tumors from MRI images, or the fuzzy approach is used to separate normal cells from abnormal cells. For tumor segmentation, great techniques of semi-automatic and automatic segmentation are used [2]. The improvement and progress in e-health and medical technologies help clinical experts to simplify more useful e-health care systems to the patients. Convolutional neural networks are designed to emulate the neural connectivity found in the brain’s visual cortex; this topic is inspired by biological processes. The advancement in technology has made significant contributions to the field of medicine. The use of augmented reality during surgery is one of these aids. A report from [11] described the use of a wearable augmented reality platform in neurosurgery. CNNs are used in different categories, such as image and video recognition, recommender systems, image classification, medical image analysis, and natural language processing (NLP). The focus of this survey is on this method and its related concepts. For image analysis and processing, CNN models are becoming more impressive, and they are increasingly able to break previous state-of-the-art classical machine learning algorithms.

## 2. Background

In this section, we review the subjects related to the topic of this study. First, we explore the importance of segmentation and classification in medical images and the problem of identifying brain tumors in MRI images. Secondly, we investigate three areas in artificial intelligence, including deep learning, neural network and machine learning. Third, we explore some applications of deep learning for medical imaging and combine them with CNN in image processing.

### 2.1. MRI Images—Segmentation and Classification

To segment, the region of interest (ROI) in image processing methods uses image segmentation and classification. In understanding images, extracting features, analyzing and interpreting images, image segmentation and classification have fundamental roles. A method to find the region of interest (ROI) or dividing the image into different regions or areas is medical image segmentation.

Failure to identify the exact location of the brain tumor leads to an incomplete, improper evacuation of the tumor, which causes the tumor to regrow and metastasize. These cases increase the risk of death. Image processing methods can be used to prevent and minimize this issue. For MR images, manual, semi-automatic or fully automatic techniques can be used. In medical image processing, manual techniques are time consuming and not as accurate as semi-automatic or fully automatic techniques. In addition, the design of a fully automated and effective classification still needs a second look because medical problems are related to human life and expert opinions are very decisive. Researchers have proposed several methods to develop such knowledge bases and, thus, the ability of tumor detection systems. MRI scanning is the most popular and general technique in neurology for imaging detailed specifications of the brain and other cranial structures. MRI can reveal flowing blood and hidden vascular malfunctions. The MRI scan is also beneficial and helpful for other brain-relevant diseases, such as Alzheimer’s disease [12], Parkinson’s disorder [13], and dementia [14]. The effect of COVID-19 on brain tissue was also investigated in MRI images in [15,16], along with many more diseases. There are various datasets available for training and testing purposes. In Table 2, the common datasets used in MR image segmentation are given.

An automatic model can solve partially this problem, for instance, we can use the abnormality and object detection methods. The efficiency of automatic techniques belongs just to the knowledge databases in the absence of experts. Researchers have improved many methods to use automatic techniques and knowledge databases to improve the capability of tumor detection systems [3].

### 2.2. AI techniques

#### 2.2.1. Deep Learning

Deep learning shows machine learning methods with multiple levels of representation. It consists of several layers, and the input of the layer is a representation from previous levels; with this structure, very complex features and inferences can be learned. A great deal of attention has been given to deep learning over the past several years for many applications in different areas, such as anomaly detection, image or object detection, pattern recognition, natural language processing. Deep learning can achieve great success in applications such as anomaly detection, image detection, pattern recognition, and natural language processing. Convolutional neural networks (CNNs), pre-trained unsupervised networks (PUNs), and recurrent/recursive neural networks (RNNs) are three different categories of deep learning.

**Figure 2:**
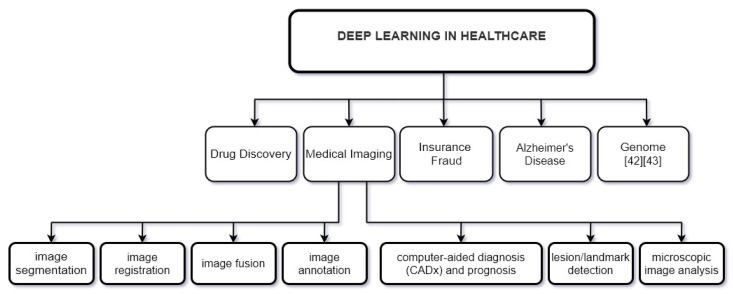
Deep learning in healthcare.

Deep learning in the healthcare system equips doctors and experts to analyze any disease more accurately and helps them in implementing treatment and improve decision making. In Figure 2, some usages of deep learning in healthcare are shown [34,35,36,37,38,39,40,41,42,43].

#### 2.2.2. Neural Network

Neural networks are chains of algorithms that mimic the operations of a human brain to recognize relationships between vast amounts of data. In recent years, DL and neural network (NN) can be superior to classical methods in object recognition methods. NNs can learn complex hierarchical representations of images because they have a strong representational power and thus, are more and more representing abstract concepts. They are strong at generalizing never-seen data. This feature empowers them to recognize a multitude of different objects whose appearance also varies greatly. For neuroscientists, a new approach for complex behaviors, heterogeneous neural activity in neural systems is provided by NNs. The benefit of neural networks is the facility of end-to-end training, and the action that neural networks generalize never seen data very powerful.

#### 2.2.3. Machine Learning and Image Processing

Machine learning is a natural outgrowth of the intersection of computer science and statistics. The learning in machine learning happens via independent optimization of internal components, which are called parameters. Machine learning methods need careful engineering and expertise in domain knowledge to design feature extractors: So Yann LeCun designed and introduced a convolution neural network (CNN) that can learn to extract features automatically. The development of machine learning and soft computing approaches have created a remarkable impact in medical imaging too [44]. The efficiency of machine learning methods is related to the choice of data features on which they are applied.

The value of machine learning for radiomic features extraction in images is idiomatic, and was first introduced by Lambin in [45]. They described the solid cancer limitation, which gives huge potential for medical imaging and extracting features. Radiomics can extract wide amounts of image features from radiographic images and addresses this problem but needs better validation in multi-centric settings and the lab. Usually, radiomic features determine one scalar value to describe a whole three-dimensional (3D) tumor volume. Some features have connections with outcomes that can be fed into a classifier; the decision tree is an example of a useful classifier. ML defines the main aspects that generate the greatest predictive ability.

### 2.3. Deep Learning Applications

#### 2.3.1. Anomaly Detection

In analyzing data, we can identify entities which are not similar to others, known as anomalies and also called outliers. Deep learning-based anomaly detection algorithms have become popular recently. In deep anomaly detection (DAD), learning methods are based on hierarchical discriminative features from data [46]. For both conventional and deep learning-based algorithms, a challenge is posed due to the absence of well-defined representative normal and anomalous border situations. Anomalies can be categorized as point anomalies, contextual anomalies, and collective anomalies. In anomaly detection, we have two types of data: sequential and non-sequential. Sequential data include video, speech, time series data, text (natural language); deep anomaly detection methods for these types are CNN, RNN, and LSTM. Non-sequential data include image, sensor, and other types of data; DAD methods for these types are CNN, AE and its variants. In addition, classification based on the type of deep learning models for anomaly detection can be (1) supervised, (2) semi-supervised, (3) unsupervised deep anomaly detection, (4) hybrid, and (5) one-class neural networks [46].

#### 2.3.2. Object Detection

Object detection is mostly used in video analysis and image understanding. For the semantic perception of images and videos, object detection can present valuable information. Additionally, in fields such as image classification, human behavior analysis and face recognition, we can use object detection.

Generic object detection aims to locate and classify images and label them with rectangular bounding boxes to show the confidence of the entity [47]. So, CNN is one of the powerful ways to determine details. CNN is used in two aspects: CNN-based deep feature extraction and classification and localization. In object detection, CNN has some advantages compared to traditional methods, thus, these advantages, including video analysis, pedestrian detection, face recognition and image classification, are some examples of CNN research fields.

#### 2.3.3. Pattern Recognition

In pattern recognition (PR), CNN has had significant success; for example, in an experimental realization of PR with CNN, for facial expression or emotion recognition, CNN design can be excellent. In the neuron model of PR, the multi-layer hierarchical network, there are forward and backward connections between cells, and CNN has good achievements. CNN is useful in text data, time-series data, and sequence input data. One of CNN’s abilities is to reduce data dimensionality, and the capability to classify in one network structure is a notable advantage of CNN.

#### 2.3.4. Natural Language Processing

Natural language processing (NLP) can be named as one of the aspects of deep learning; In some cases, CNN is used in NLP, for example, in the case of utilizing CNNs for NLP inputs are sentences that they show as matrices. Each row of matrices are consist of a language element such as a words or a characters. To extract fixed features, CNN operation is good [48]. CNN achieves results in natural language processing applications because it can reduce most traditional problems. The convolutional architecture for language tasks is to apply a nonlinear (learned) function.

Natural language processing can be used for the classification of MRI reports. Unstructured text data, such as nursing records, reception reports, and discharge summaries, that are part of medical reports can be studied by NLP. NLP tools can be applied in a rule-based method to analyze the meaning of texts; moreover, several reports have used NLP to predict the progression of cancer or to classify breast pathology by analyzing free-text radiology reports [49,50].

## 3. Literature Review

This section overviews existing studies on tumor detection from a medical aspect. In Section 3.3, we review existing surveys on CNN. In Section 3.4, the three top CNN models which we use in our topic are presented. In Section 3.5, some hybrid techniques in classification with their examples are named.

### 3.1. Tumor Detection—Classic Approach

Segmentation, classification for tumor detection and localization based on MR images, is one of the concepts of medical image processing. Necessary specifications of brain tumor types and the identification of different segmentation and classification techniques that are successful for the detection of a range of brain diseases are presented in [3]. In this survey, the most relevant strategies, methods, working rules, preferences, constraints on MR images are covered. Designing an automatic algorithm to detect the brain tumor from MRI by artificial neural networks is studied in [51]; in this article set of image segmentation algorithms, feature extraction is proposed. The proposed algorithm was successfully tested and achieved the best results with an accuracy of 99% and sensitivity of 97.9%. A brain tumor detection system based on machine learning algorithms is proposed in [52], using gray level co-occurrence matrix (GLCM) to extract the texture-based features. In total, 212 samples of brain MR images are considered, and in the classification, perception and the Naive Bayes machine learning algorithm are used. The detection of tumors based on the programmed division strategy based on CNN is studied in [53]. For the detection of tumors from MRI images, the MATLAB tool is used by performing SVM classification. The processes and techniques used in detecting tumors based on medical imaging results are reviewed in [54]. A fully automatic brain tumor detection and segmentation method using the U-Net-based deep convolution networks is presented in [55]; they used BraTS 2015. The brain tumor segmentation model without manual interference is presented in this study. A mix of hand-crafted and deep learning features for segmentation image is presented in [56]; they used the grab–cut method for accurate segmentation. An automatic system for tumor extraction and classification from MRI based on marker-based watershed segmentation and features selection is developed in [57]. For diagnosis of the hardest brain tumor situation, in Ref, [9], the authors used deep CNN. They used MATLAB software, and their database consists of 1258 MRI images of 60 patients. The result of the study gained 96% accuracy.

### 3.2. Deep Learning and AI for Medical Imaging

AI methods for imaging methods have many suggestions to share image data better on different platforms [58]. Artificial intelligence improves radiologists’ diagnoses of malignant and benign tumors using MRI images in breast lesions [59]. AI allows radiologists to better diagnose, which may improve the patient’s therapy and cure. A high-performance algorithm to discover and characterize the presence of a meniscus tear on magnetic resonance imaging examination (MRI) of the knee is built in [60]. Analyzing the literature on artificial intelligence (AI) and radiomics, including all medical imaging modalities, for oncological and non-oncological applications for routine medical application is reviewed in [61]. A general outlook of deep learning-based MRI image processing and analysis is presented in [62]. A deep learning algorithm that can exactly diagnosis breast cancer on screening mammograms and uses an end-to-end training approach with a deep learning algorithm is developed in [63]. The main deep learning concepts which are related to medical image analysis are presented in [39].

### 3.3. CNN for Medical Imaging

Several tutorials and surveys have been published with a focus on convolutional neural networks and their challenges. Zhu Wenwu and his coworker in [64] in their book completely explained CNN structure with layers, and they explained CNN applications and architectures. They also described forwarding and backward propagation in CNN. Sergio Pereira et al. in [65], used normalization as a pre-processing step based on CNN. They worked on BraTS 2013 and 2015, and proposed an automatic segmentation method based on CNN. Darko Zikic et al. [66] studied the possibility to directly apply convolutional neural networks to the segmentation of brain tumor tissues. The matter is that the input to the network used multi-channel intensity information from a small patch around each point to be labeled. Only standard intensity pre-processing was applied to the input data to account for the scanner differences. No post-processing was applied to the output of the CNN. Jose Bernal et al. in their survey [67] presented a review of CNN techniques that are focused on architectures, pre-processing, data preparation and post-processing strategies in MRI images analysis. They reported how different CNN architectures have evolved, and also discussed state-of-the-art strategies.

Amin Kabir et al. in [8] used a method that derives from the combination of CNN and the genetic algorithm. They proposed to noninvasively classify different grades of glioma using MRI images to reduce the variance of prediction error, and utilized bagging as an ensemble algorithm. Jin Liu and Min Li [1] provided an overview of the concept of the brain tumor segmentation methods, object detection, registration and other tasks; the preprocessing methods for MRI-based brain tumor segmentation were introduced. A 3D fully connected conditional random field, which effectively removes false positives, was used in [7]. Additionally, for automatic lesion segmentation, they presented a 3D CNN architecture.

A dual-force training strategy was proposed in [68] to explicitly encourage deep models to learn high-quality multi-level features. The main point in [69] is a fully convolutional network whose input size is optional and generates a correspondingly sized output with effective inference and learning. Havaei et al. [4] proposed FCNN to segment and test images slice by slice. Additionally, a two-phase training scheme was proposed to deal with the class imbalance problem. Menz et al. [19] represented the multimodal brain tumor image segmentation. This method can be categorized as generative or discriminative. Fritscher et al. [70] presented a CNN for 3D-based deep learning components, which consists of three convolutional passes. A DCNN for multi-modal images was presented in [71]. Three architectures were proposed, whose patch (input) sizes are different. In addition, it proved that the size of the patch and the size of the convolutional filter affect the results when we use a patch way for segmenting brain tumors.

### 3.4. Modeling in CNN

The cascaded-CNN (C-CNN) is a novel deep learning architecture comprised of multiple CNNs [72]. CNN architectures are different in the depth of the network and the number of users. In continuation, three models based on the Multimodal Brain Tumor Segmentation Challenge (BraTS) are introduced.

#### 3.4.1. Ensembles of Multiple Models and Architectures (EMMA)

This algorithm in the BraTS 2017 competition can obtain the first position between more than 50 teams. The performance of this algorithm is supreme because it combines multiple configured and trained CNN models [73]. Deep-Medic is the first employed architecture in this model; Deep-Medic is the 11-layers deep, multi-scale 3D CNN for brain lesion segmentation. EMMA can integrate two versions of 3D-Unet [73]. In the testing steps, any segmentation model segments images and outputs its class-confidence maps. EMMA is a deep learning model which can be run with great performance.

#### 3.4.2. CNN-Based Segmentation of Medical Imaging Data

The performance of this model is similar to the U-Net CNN architecture with two rectifications: (1) merging multiple segmentation maps created at different scales, and (2) consigning feature maps from one stage of the network to another by using element-wise summation [73]. The CNN-based method with three-dimensional filters is demonstrated and applied to hand and brain MRI based on medical imaging data in [74]. In addition, two modifications to an existing CNN architecture are discussed, along with methods for addressing the aforementioned challenges. This model can achieve the first rank in BraTS 2015 and ischemic stroke lesion segmentation (ISLES).

#### 3.4.3. Auto Encoder Regularization Based

This algorithm was introduced by Andriy Myronenko. This algorithm for extracting image features uses an encoder–decoder-based CNN architecture. For tumor subregion segmentation from 3D MRIs based on encoder–decoder architecture, a semantic segmentation network is used in [75]. The current approach won 1st place in the BraTS 2018 challenge.

### 3.5. Hybrid Techniques in Classification

Hybrid techniques integrate two or more techniques to obtain better results compared to individual techniques. In Table 3, some examples and usage of these techniques in analyzing MR images are given.

## 4. Learning Methods and Related Concepts

This section discusses and contrasts the key concepts of learning methods and related concepts. We review the CAD system and some usages which are based on machine learning. We discuss segmentation and classification. The last subsection is about semantic segmentation.

In medical imaging in training data for machine learning and deep learning algorithms, big data plays a critical role [3]; the number of layers or depth in the ANN is defined by DL. Generally, DL has a connection to CNN to identify and extract features directly from images [84].

### 4.1. Method of Learning

Generally, three types of learning algorithms exist in both machine learning and deep learning, based on the labels of the training samples they are categorized into: supervised learning, semi-supervised learning, and unsupervised learning algorithms.

#### 4.1.1. Supervised Learning

Any model or example in supervised learning contains two parts: features and labels. In convolutional neural networks (CNNs), supervised learning is related to labeled data. In each sample we have lots of data and labeling of each data can be very time consuming and costly for solving this problems and it is one the problems in this method.

#### 4.1.2. Unsupervised Learning

There is no label in unsupervised learning; it consists of a set of observations for each example. Discovering the relationships between samples is the main purpose of unsupervised learning. The clustering algorithm is a representative method of unsupervised learning [1]; this model can be shorter. In Ref. [85], unsupervised learning is applied in a CNN, and selective unsupervised feature learning is explained with a convolutional neural network.

#### 4.1.3. Semi-Supervised Learning

Semi-supervised learning is used for both labeled data and unlabeled data for training, and it consists of supervised and unsupervised learning. It was developed because the labeling of data is very expensive, time consuming and impossible in some applications. A semi-supervised dataset consists of unclassified training data with small amounts of classified data, and they have two important advantages: first, they have a few classified data, so they are more accurate than unsupervised models; second, compared to supervised learning, they are less difficult and time intensive. One of the purposes of the semi-supervised method is solving the problem of limited labeled samples. For the classification of a hyperspectral image, a new semi-supervised convolutional neural network is presented in [86].

### 4.2. Review of CAD System

**Figure 3:**
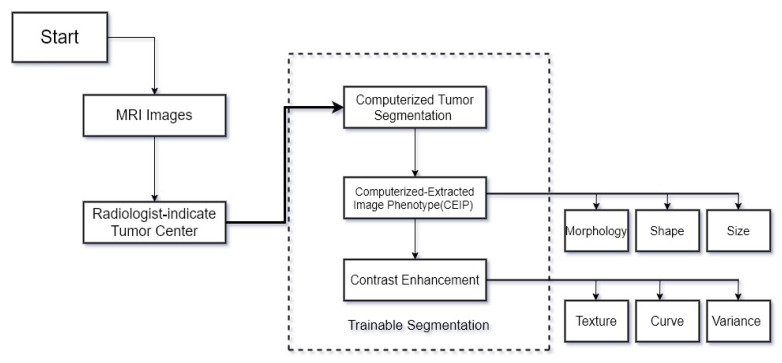
Flow diagram of the CAD system.

Computer-aided diagnosis is a concept that works based on both physicians and computer vision, and the difference with computer diagnosis is that it does not work entirely based on computer algorithms. CAD system diagnosis is considered a second opinion and makes decisions based on [87]. Computer-aided diagnosis (CAD) systems help the radiologists to visualize the imaging modality in a much better way as compared to the naked eye. In Figure 3, a flow diagram of a CAD system in MRI images is shown. This system is based on machine learning. With such a system, the disease is diagnosed much more easily and accurately. This system receives a lot of input, which is why it easily detects damaged brain tissue in MRI images. The main purpose of the CAD system is to assist radiologists in interpreting images as a second opinion. CAD increases the accuracy of the radiologist’s diagnosis and generally greatly avoids possible errors in the diagnosis.

#### Example of Studied Based on CAD System

CAD systems assisted in the diagnosis of the early treatment of breast cancer in [88]. Breast cancer was extracted from different image techniques. CAD systems are used in clinics, regardless of the method used. The CAD system improves clinical performance and reduces misunderstandings of images that may cause the correct treatment to be ignored. Alzheimer’s disease is one of the most common diseases of the past decade. The CAD system based on independent component analysis (ICA) for the early detection of Alzheimer is proposed in [37]. In this work, CAD is built in two stages; firstly, feature extraction is based on independent component analysis (ICA) and, secondly, support vector machine (SVM) training and classification. Early detection of cancer can improve the patient’s survival. Lee and et al. in [89] studied the CAD system for the detection of cancer, namely, breast cancer, prostate cancer, lung cancer and skin cancer. We can say that this study encourages doctors to collaborate with CAD systems for cancer detection because CAD systems extract biometric information. The size and thickness of the organs and tissues are biometric information and can help identify the exact location of cancer cells. Biometric information help experts to specify the types of skin cancer and monitor its propagation on the skin surface.

### 4.3. Segmentation and Classification

For brain tumor studies, segmentation and classification are the most important and the most challenging topics in image processing, so this survey is focused on this topic. The aim of segmentation is to partition images into several parts. Additionally, segmentation can be performed according to tissue types, functional areas, etc. Different types are available for tumor segmentation: manual, semi-automatic, and fully automatic. In a fully automatic method, computers do everything; usually, this method is combined with artificial intelligence, which uses machine learning algorithms and for which CAD systems are in action. Machine learning automates the analysis and recognition of a medical image. The most popular unsupervised segmentation method for brain tumor is clustering because of group data with certain similarity criteria [90].

A huge amount of data are produced by MRI images, so automatic approaches are needed for MRI image segmentation. Before the segmentation, we need to preprocess images, which appoint segmentation purposes. Preprocessing comprises some steps that include de-noising, skull-stripping, intensity, normalization, etc.

Generally, segmentation is performed manually, which is time consuming and tedious for radiologists and has limitations for an objective quantitative analysis. Manual classification is based on the personal opinion and experiences of the evaluators. Because of this, it can have errors. Therefore, fully automated and accurate systems for brain tumor segmentation are highly desirable in practice. Segmentation has important aspects in medical imaging matters, for example, during treatment to monitor tumor growth or contraction in patients; for tumor volume measurements and surgery, it can identify areas that have tumors. Some algorithms that mostly use in brain tumor segmentation are based on classification or clustering methods, such as fuzzy c-means (FCM), k-means, Markov random fields (MRF), Bayes, artificial neural networks (ANN), and support vector machines (SVM).

Brain tumor segmentation is divided into two categories: generative models and discriminative models. Generative models are based on domain-specific knowledge about the appearance of both healthy and tumor tissues. Markov random field (MRF) and conditional random fields are two examples of generative models. Discriminative models do not need any domain knowledge, as they can learn the connection between image intensities and segmentation labels directly [19]; CNN is a type of discriminative model. These models can handle the segmentation problem in a pattern classification setting.

The deep convolutional neural network (DCNN) can learn features automatically and obtain complex function mapping, so it is used in complex image processing. This method is categorized in two ways: the patch-based way and end-to-end way. The input of the network for the path-based way is patches with usually a fixed and odd size, and the output is the class of its central pixel [90].

### 4.4. Semantic Segmentation

Image segmentation is one of the computer vision functions; in image segmentation, labels indicate what each particular part of the images represents. The aim of semantic segmentation is to label each pixel of an image with a corresponding class of what is being represented. The category of each pixel is determined in semantic segmentation.

## 5. Convolutional Neural Networks

This section focuses on the importance of CNN and the studies that are leveraging neural networks to tackle the problem of tumor detection. A comparison of different CNN architectures is reviewed in this section, and we talk about the usages of CNN methods in medicine.

### 5.1. Importance of CNN

Traditional neural networks are called the multilayer perceptron (MLP). MLP has drawbacks, such as using a perceptron for each input, which becomes uncontrollable for large-weight images. Another problem is the different response of MLP to an input (images) and its modified version. MLP cannot be a good option for image processing because spatial information is lost when the image is flattened into an MLP. One of the most effective methods of deep learning for image analysis to date, which has made noteworthy improvements in the image processing field, is convolutional neural networks. (CNNs/ConvNets) have many important achievements in resolving complex problems of machine learning. In neural networks, CNN is one of the main classes. CNN image classifications take input images, process these, and classify them with certain categories, e.g., cat and dog. The role of CNN is to decrease an image into a shape that is easier to process without losing any acute features, which are needed for a beneficent prediction. CNN has a powerful capability in processing images and learning features. CNN has a critical role in different deep neural network applications [64].

**Figure 4:**
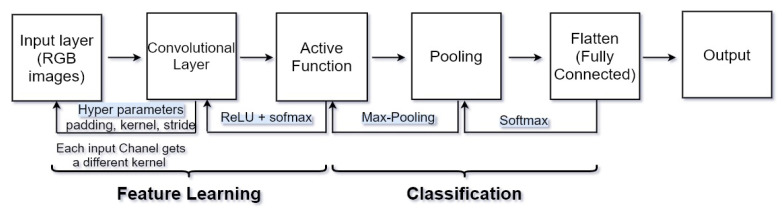
CNN layers, consist of 7 layers, input: [[CONV to RELU] × 2 to pool] × 3 to FC.

CNN has recently shown noted performance in computer vision for image segmentation and classification tasks; CNNs can learn the most useful features automatically. Convolutional layers that each input passes are kernel, pooling, fully connected, and SoftMax function. Figure 4 shows a complete CNN stream of processing an input image and classifying objects based on values. CNN contains many layers that transform their input with convolution filters of a small extent. Convolutional (sets of learnable filters), pooling (used to reduce overfitting and reduce image size) and fully connected (used to mix spatial and channel features together) are the three main layers of a convolutional network. The CNN layers are shown in Figure 4.

Convolution operators are used in most layers of these networks. In recent years, CNNs were applied in the segmentation of MS lesions, cerebral micro-bleeds and deep brain anatomical structures. High computational costs can be solved by convolutional networks, and this feature is very important since thousands of MRI images with different qualities and types are used for diagnosis, so CNN is used to classify brain tumor images. CNN can extract features automatically and further decrease the dimensions. CNN has performed well in processing medical images using deep neural networks. Deep learning algorithms, remarkably convolutional networks, have quickly become a methodology of election for analyzing medical images.

### 5.2. U-Net and Fully Convolutional Network

CNN has two main drawbacks: (1) the network works quite slowly because of the redundancy caused by the overlapping patches, and (2) the exchange that takes place between localization accuracy and classification accuracy [91]. Additionally, large patches use more context and they are more accurate, but they reduce the accuracy of the localization. For solving these problems, fully convolutional networks were introduced. Semantic information with appearance information are combined in FCN; FCN can produce accurate segmentation results [69]. Some good examples of FCN in achieving good results in medical image segmentation are shown in Refs. [92,93,94]. FCN is an instance of dense prediction networks.

U-shaped architecture, named U-Net, was developed by Ronneberger [95]. U-Net is a fully convolutional network that consists of a contracting path and an expansive path. The contracting path works as a feature extractor, and it follows the generic architecture of a convolutional network. The expansive path increases resolution by utilizing up-convolution; the ability of this network is obtaining the final segmentation results with only one training session [91]. U-Net gradually presents feature maps by connecting its encoding layers to its decoding layers of the same resolution for better perception and utilizes multi-level features [68]. We can say that FCN and U-Net are dense prediction networks, and DeepMedic in another example of this network [7]. No pooling operations exist in DeepMedic, and the decrease in feature map size is realized by canceling padding operations in convolutional layers [68]. We can say most of the current methods correlate multi-modality MRI data together as input. Table 4 provides a comparison of CNN modalities.

### 5.3. Comparison of Different CNN Architectures

The most common CNN architectures are LeNet, AlexNet, VGGNet, GoogLeNet, ResNet, and ZFNet. They are implemented based on CNN. U-Net, SegNet and ResNet18 are the most popular CNNs for image segmentation [98].

LeNet, developed by Yann LeCun in the 1990s [99], is the first prosperous application of convolutional networks. Some usages of LeNet architecture are reading zip codes, digits, etc. One model of LeNet consists of five-layer CNN, which is called LeNet-5, and it can gain 99.2% accuracy on a single character recognition. The AlexNet, developed by Alex Krizhevsky [96], was the first convolutional network to become popular. The first five layers of AlexNet are convolutional layers, and the last three layers are fully connected layers, which contain eight main layers in total. To increase the speed and accuracy of AlexNet, ReLU is used. Microsoft Research in [100] proposed the residual neural network (ResNet). In ResNet, instead of unreferenced functions, layers are reformulated while learning residual functions. Residual networks have higher accuracy from increasing network depth, and the optimization of this network is easier. GoogLeNet was designed by Szegedy et al. [101], and it contains 22 layers. In comparison with AlexNet, it is much deeper. GoogLeNet contains 4 million parameters, and AlexNet contains 60 million parameters. One of the most used versions of GoogLeNet is Inception-v4. In Table 5, CNN architectures are compared with each other, and Table 6 provides some examples of CNN’s architectures in medicine.

To purify the prediction outcomes of CNNs in network architectures, different post-processing methods were proposed. For instance, in [7], 3D-CRF was chosen for post-processing, which cures segmentation results by minimizing the Gibbs energy of every voxel. In addition, Havaei [4] presented clear predictions which are unusual in regions close to the skull according to the intensities of the voxels and the volume of the tumor area. A more complex post-processing pipeline is presented in [116], which is dependent on the voxel intensity, volume of the predicted area, etc. Setio et al. [117] used multi-view convolutional networks for pulmonary nodule detection; their network architecture is composed of multi-stream 2D CNNs.

### 5.4. Usages of CNN Methods in Medicine

In the U.S., breast cancer is the second major cause of cancer-related death. With mammography screening, mortality from breast cancer is reduced. The CAD system is used in mammography screening to improve the guessing accuracy. A modern CNN in the input has convolutional layers, and the output has one or more fully connected (FC) layers. In the paper of Shen et al. [63] was compared to methods of CNN, that are VGG and residual (Resnet) networks.

The visual geometry group (VGG) block is a stack of several 3 × 3 convolutional layers with 2 × 2 max pooling to reduce the feature map. The quality of the patch classifiers is important in the final classifiers’ results. Colorectal cancer (CRC) is the third most commonly diagnosed cancer [118]. To recognize and segment the exact location of tumors in CRC, MRI has good advantages. For the extraction of features from the colorectal tumor image, VGG-16 was used as the main model in [91], and for classification and localization information, five side-output blocks were used. In Table 7, the CNN methods in medicine are discussed.

#### Usages of CNN in E-Health

In recent years, CNN has been combined with the internet of things in wearable sensors toward improvement in the healthcare system. Progressive medical methods, such as telemedicine, image diagnosis, disease prediction, healthcare and so on, with the development of wearable sensors have been introduced. To mange daily life activities and healthcare, Ref. [120] focused on the wearable smart watch. In this paper, they used CNN for their target. A good example for the wearable sensor is presented in [121], a lightweight human bowel sounds (BSs) application.The recognizer is based on CNNs, and it is proposed for wearable systems. A lightweight CNN was used in [122] for the classification of a multivariate electroencephalogram (EEG). An online and accurate analysis for big data which are related to the brain is presented in this paper. Potentials of IoT technologies in brain healthcare are presented in [122]. Based on the collaborative machine learning approach in the field of IoT eHealth architecture, Ref. [123] reviewed arrhythmia detection by the use of CNN. The classification of tongue color based on CNN was studied in [124]; for training and testing images, they used CNN. Their experimental results showed that as the dataset increases, the accuracy becomes higher. For detecting tuberculosis in chest X-ray imaging, CNN was used in [125].

## 6. Neural Networks and Beyond

This section details some of the existing methods which are related to CNN and combined with other methods to obtain a better result and fix some shortcomings in image processing and learning. We focus on examining existing methods for processing brain MRI images. In future work, some of these methods will be further explored.

### 6.1. Representation Learning

Representation learning enables systems to assign convenient features for a given task automatically. Additionally, it can be supervised like k-means or unsupervised like a neural network. In the pre-trained architectures, the transfer of knowledge existed. Transfer learning is one of the pre-trained architectures and one of the important aspects of CNN. Pre-trained CNN should be successful in different applications, such as the 3D FCNN trained on subcortical brain structures in [5]. The use of transfer learning for subcortical structure segmentation to dominate the domain shift problem is presented by Kushiber et al. [126]. In medical image analysis, if we do not want to use a large amount of data, the transfer learning strategy is a good choice to dominate the request for deep learning methods for a large amount of data. One of the goals of transfer learning is to use only a few images for training to adapt a network that performs well in a new domain. To achieve better results from deep learning, using transfer learning is a good method which can detect normal and abnormal brain tissues based on MRI images. For heterogeneous brain tumor segmentation, Ref. [127] is an example of automatic end-to-end trainable architecture. An exact and fully automatic system, with minimum pre-processing, for brain tumor classification is presented in [128]; this proposed system for extracting features from brain MRI images used the meaning of deep transfer learning.

### 6.2. Pre-Trained Unsupervised Networks

A model that is trained and created on a big dataset to solve a problem like ours is named a pre-trained model. Complex models are prone to overfitting, so training deep feed-forward neural networks can be difficult. A deep belief network or a deep autoencoder are examples of unsupervised pre-training. Optimizing and overfitting issues can be handled with this method. Using features from a pre-trained network can give results that are comparable with those of a network that, with important effort and resources, is trained from scratch for the domain in focus. The feed-forward neural network architecture consists of a set of neuron-like “units”; each unit computes a simple function of any inputs, and the structure is based on supervised learning. Data generation and feature exploitation are major applications in deep learning. Usually, the limitation is the training data. To provide the large dataset that is needed to train the network, different techniques are used to increase the early dataset [129]. To improve the learning model by generating synthetic data based on the main dataset, we can use generative adversarial networks (GANs) and autoencoders, which are the most progressive architectures, and both of them are unsupervised pre-trained networks. An unsupervised pre-trained network for training each hidden layer uses unsupervised learning because the results are more accurate for a dataset. In an unsupervised learning algorithm, each input layer uses a previously trained layer as input, and this input only trains one at a time. After applying the pre-trained network on each layer, supervised learning is used for a fine-tuning step on the whole network [130].

#### 6.2.1. Autoencoders

Autoencoders for transfer learning have a basic role in unsupervised learning and deep architectures. Copying input to output is the main goal of autoencoders. An autoencoder is generally a type of artificial neural network for learning effective data encoding in an unsupervised way. This network has two main objects: encoder and decoder. An encoder is a part of network that compress the input into a hidden-space representation. Autoencoders are able to automatically learn from data samples. The autoencoder can aim the most important features of the training data by training an under-complete representation. One of the weaknesses of the autoencoder is the increase in capacity, which, if exceeded, distributes the data without providing useful information. In autoencoders where the input is the same as the output, we use unsupervised learning to learn a representation to reduce the dimensions [129].

#### 6.2.2. Generative Adversarial Networks

**Figure 5:**
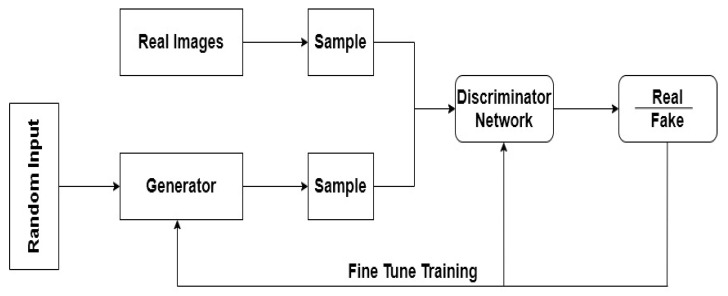
A representation of GAN network.

Generative adversarial networks (GANs) are an instance of a network that uses unsupervised learning to train two models in parallel. One of the fundamental goals of generative adversarial networks (GANs) is the training of two deep learning models together. These networks consist of two models: generator (G) and discriminator (D). The generator generates new samples or examples, and the discriminator classifies a particular model that originates from the training data or the generator. In the network, when the amount of data is smaller than normal, using GAN is a solution. The aim of the generator is to be able to create a fake output that is most similar to the real output [131]. The output generated by the generator must be so close to the real one that it is not possible to distinguish the fake data from the real data. GANs have three main phases: (1) in the first phase, the generator chooses accidental numbers and returns an image. (2) In the second step, we have two probabilities, 0 and 1; both real and fake images are taken by the discriminator and return probabilities. If the output is close to 0, the data from the generator are fake, but if an output is close to 1, the data are real. (3) In the third step, the discriminator network provides feedback to the generator to train it and improve its output. We can use GANs to improve the resolution of images or to create photos based on an exact caption description. GAN and CNN are used for the classification of hyperspectral images (HSIs) in [132]. A novel semi-supervised algorithm for the classification of hyperspectral data by training a customized GAN for hyperspectral data is proposed in [133]. A deep neural network with generative adversarial network pre-training for brain tumor classification based on MRI images is presented in [134]. To pre-train the deep network, it was adopted as a discriminator in a GAN architecture, and the overall performance was intensely increased. In Figure 5, a GAN network is presented.

#### 6.2.3. Deep Belief Networks

A deep belief network is a link established between each network of connected neural networks in different combinations in series with one another. Deep belief networks are part of the category of unsupervised learning to generate output. They are composed of binary latent variables, and they contain both undirected layers and directed layers. In deep belief networks, each layer learns the entire input. Generally, a belief network includes binary unit layers which are generated accidentally. Each connected layer is assigned a weight function, and the area of these binary units is from “0” to “1”. In this network, learning occurs layer by layer so we can define how a variable in one layer can interact with those variables in another level. The architecture of a deep belief network is like a stack of restricted Boltzmann machines (RBMs). A RBM is an accidental recurrent neural network, where the nodes in each layer are connected to all the nodes in the prior and next layers. We can use deep belief networks in image recognition, video recognition, and motion-capture data. For learning the unlabeled features in the classification of brain tumors, an unsupervised deep belief network (DBN) on MRI images is used in [135]. In this research, the authors analyzed various images of the BraTS 2015 and achieved 91.6 percent accuracy on its classifications. An automatic brain tumor segmentation method on the BraTS 2012 and 2013 datasets based on deep belief networks (DBNs) and pathological knowledge is proposed in [136]. A convolutional deep belief network (CDBN) is used to pre-train the weights of the CNN system in [137] to detect pathological voices.

### 6.3. Recurrent Neural Networks

Recurrent neural (RNN) networks are a class of deep learning. Where the information sequence is critical, these networks have worked well, due to their ability to process serial data. Therefore they can be used in natural language processing (NLP), video analysis, image captioning, speech synthesis and machine translation. For every single element of a sequence, RNNs have a monotonous task; in this network, output is related to the prior computations so they are called ’recurrent’. Two of the most common RNN architectures are: (1) Bidirectional RNN—in this network, the output is related to both the previous and subsequent results. (2) Deep RNN—in this network, there are several layers at each step, which makes learning more accurate. Theoretical RNN can recall information for long periods, but practically this is not always the case [129].

## 7. Discussion

This section includes the identified challenges and existing solutions in the topic of this survey as well as a shortlist of some future directions.

### 7.1. Challenges and Solutions

Some known technical challenges and suggestions are provided in Table 8 which are mentioned in different surveys.In addition, existing challenges in the topic of using deep learning for medical imaging for tumor detection can be considered from several perspectives as follows.

#### 7.1.1. First Time Consuming

Manual annotation is time consuming. One of the requirements for training neural networks is to have sufficient annotated training data. Training on a huge amount of data is very calculation intensive; even on powerful graphics cards, it takes several days. During the conclusion, the fully trained networks generally perform the analysis of an image quickly. The sensibility of DL models to hyperparameters is that they have to be set correctly to obtain optimal results. Manually tuning these hyperparameters belongs to the domain knowledge of their effect on the generalization of the network [139] and is time consuming. Remarkable improvement has been done in the automatic tuning of hyperparameters for DL models, but this process is still computationally intensive.

#### 7.1.2. Accuracy of Diagnosis

One of the reasons for using artificial intelligence technologies in medicine is to increase the power of diagnosis and minimize human error. In cases where combined methods have been used, this accuracy has increased even more, for instance, in [140]. We have shown that using hybrid self-supervised/supervised learning and multi-modality training data (2D and 3D) on over 105,000,000 images can increase accuracy by 6–8%.

#### 7.1.3. Need Second Opinion

For the design and implementation of fully automatic and effective tumor detection, the segmentation and classification system presents many challenges for physicians and radiologists. Such a system always demands a second opinion because it is related to human life. On the other hand, such a system could improve the accuracy, speed and performance of detection. The motivation of medical images is to localize the tumor region to simplify the operation medically.

#### 7.1.4. Complexity of Computational

Some challenges remain for detection techniques. The complexity of computation in a study of multi-modal MRI image modalities at a single instance is one of the challenges in imaging techniques.

#### 7.1.5. User Rights

In medical research, often, we face the issue of the availability of data and medical information. To respect user privacy, clinics and hospitals are not able to share patient information. Although this important aspect is not in the scope of this survey, we do believe that having a frame to securely share this type of data is a hot topic to be considered as a future direction of this research.

### 7.2. Future Directions

We have looked at the future work in two directions: data and modeling.

#### 7.2.1. Data Perspective

Due to some problems in the field of medicine, such as not sharing patient information and the lack of data, there are some restrictions. Therefore, for this limitation, pre-train methods, as needed, can be used on existing datasets. A deep convolutional neural network is using for the multi-class classification of MRI brain tumors. In this method, the learning algorithm has two steps: pre-training and fine-tuning. Pre-training in this method is presented as an unsupervised learning approach that needs no tagged data; so, any MRI dataset can be used and aims to answer the low dataset size problem. In transfer learning, many of the pre-trained models used are based on a large CNN.

#### 7.2.2. Modeling Perspective

Time-saving and accurate models are very popular in computer vision, and transfer learning is one of the models. With transfer learning, you start learning from patterns that were learned when solving a different problem and use the previous learning to avoid starting from scratch. Therefore, it is faster and we can save time. As a result, in image classification and segmentation, it can be used in large datasets and gives more accurate results. Labeling-free brain-tumor classification that was reviewed in [141]. In this approach, 3D sequence images are used, and the pixel-wise or slice-wise labeling is not needed, which increases the speed and is more effective and efficient. In the future, this method could be applied to larger datasets.

The instance segmentation concept consists of object detection and semantic segmentation, which is a quickly growing application area of CNNs. Instance segmentation models are powerful at image detecting accurately. One of the novel methods, called Mask R-CNN, is faster than R-CNN in tumor detection [142]. Transfer learning, which focuses on transferring knowledge across domains, solves this problem. Transfer learning is sometimes used in conjunction with various CNN architectures for example in [143], four different pre-trained deep CNNs—AlexNet, ResNet18, DenseNet201, and SqueezeNet—were used transfer learning. For pneumonia detection using chest X-ray on a study on COVID-19, the authors in [144] used chest X-ray images to easily diagnose COVID-19. They used the CNN-based transfer learning BiLSTM network.With a hybrid architecture, they increased the accuracy of COVID-19 classification to 98.70%. The genetic algorithm (GA) is very efficient in solving large-scale problems and can be used to find an optimal (or near optimal) solution; in combination with CNN-based methods, they can act very efficiently in tumor classification. Self-supervised learning can work well on large volumes of data. An example of using this method is given in [140]. In this article, the authors used self-supervised learning based on contrastive learning and online clustering. Their dataset included over 1,300,000 X-rays and a dataset of over 105,000,000 multi-modality image data. They used this method for assessing chest abnormalities, and diagnosing brain metastasis, MRI, and cerebral hemorrhage on CT. One result was a 6 to 8 percent increase in accuracy. For a large amount of data, this method can be considered one of the most used methods with acceptable accuracy.

## 8. Conclusions

Saving human lives from known diseases, such as brain tumors, is one of the main concerns in modern societies. With the recent advancement in technology, medical imaging has been inspired by artificial intelligence methods, such as deep learning. These methods enable accurate analysis of large datasets in which models are trained to identify anomalies. Among many machine learning models, artificial neural networks (ANNs) are popular in image processing, such as image classification and segmentation, and many advanced models of convolutional neural networks (CNN) have been proposed in those areas. The basis step in image processing techniques is segmentation, because the goal is to extract infected region and anomalous areas from MRIs. In this study, we provide a general study on deep learning methods and other trending techniques for brain tumor segmentation and classification in the domain of magnetic resonance (MR) images. We focused on CNN in more detail and studied different architectures and their usages in medical imaging. Based on our investigation, we identified the existing gaps in the domain to provide a set of future directions on this subject. This article focused specifically on the various CNN architectures in medical image processing and their results.

## Figures and Tables

**Table 1:** List of the existing related surveys to this study.

Ref.	Year	Focus of the Survey	Description	Distinguishing Features to Our Study
[1]	2014	Brain tumor segmentation	General overview for MRI-based brain tumor segmentation methods.	Only focus on MRI-based brain tumor segmentation
[4]	2017	Brain tumor segmentation	A deep convolutional neural network for an automatic brain tumor segmentation.	Presented a fully automatic brain tumor segmentation method based on DCNN by considering different architectures and their impacts.
[5]	2018	Subcortical brain structure segmentation	A 3D-CNN for segmentation of the subcortical brain in MRI images.	Presented a method based on fully-convolutional networks, they show their performance on the ISBR dataset.
[6]	2016	Brain extraction of MR images	For extraction brain MRI images using 3D convolutional deep learning.	It is about the 3D convolutional deep learning architecture which handles an optional number of modalities for large-scale studies.
[7]	2017	Brain lesion segmentation	Brain lesion segmentation based on 3D-CNN architecture, DeepMedic.	For brain lesion segmentation presented a dual pathway 3D-CNN.
[8]	2019	Classifying glioma brain tumor	A combined method from CNN and genetic algorithm for classifying glioma brain tumor from MRI images.	They focused on a combination of genetic algorithm and CNN
[3]	2020	Brain tumor detection for MR images	Review of numbers of segmentation and classification techniques which are used in detection of brain diseases.	Mostly they discussed different types of MRI images and focused on the medical sides of brain tumor classification.
[9]	2021	Brain tumor diagnosis	Diagnosis the hardest tumor situation in radiology with Deep CNN	Using MATLAB software for processing and their database collected on 1258 MRI images from 2015 to 2020

**Table 2:** Available datasets of MR images.

Dataset	Description	Ref.	Features
BRATS	Brain Tumor Segmentation Challenge (BRATS) always focus on the evaluation of current and novel methods for brain tumors segmentation in multimodal MR images and has the dataset available from 2012 to 2020.	[17,18,19]	Fully Convolutional Neural Network (FCNN) and Conditional Random Fields (CRF) used in Brain tumor segmentation and this is based in conjunction with the MICCAI 2012 and 2013 conferences.
OASIS	Open Access Series of Imaging Studies is contained over 2000 MR sessions are collected among several ongoing projects through the WUSTL Knight ADRC	[20,21]	Diagnosis of Alzheimer’s Disease.
TCIA	The Cancer Imaging Archive (TCIA) is a big archive of cancer images and available for public download.	[22,23,24]	Prediction of head and neck cancer and Prediction of pancreatic cancer. Segmentation of brain tumors.
IBSR	The Internet Brain Segmentation Repository. Its goal is to encourage the evaluation and expansion of segmentation methods.	[6,25,26]	Segmentation of MRI images and skull stripping.
BrainWeb	It is a Simulated Brain Database.	[27,28,29]	Reconstruction of 3D MR images based on CNN and reduction of noise from MRI images and segmentation of cerebrospinal fluid and brain volume-based CNN
NBIA	National Biomedical Imaging Archive that is for in vivo images, these images are related to biomedical research community, industry, and academia with access to image archives.	[30]	Quantitative Imaging Network.
The Whole Brain Atlas	This site has dozens of real images of the brain and the Harvard Whole Brain Atlas provides you with access to PET and MRI scans of normal and diseased brains.	[31,32]	Features Extracted from brain images by CNN and Serotonin Neurons.
ISLES	Ischemic Stroke Lesion Segmentation a medical image segmentation challenge at the MICCAI 2018 and a new dataset is consist of 103 stroke patients and matches profesional segmentations.	[7,33]	Brain lesion segmentation and stroke lesion segmentation.

**Table 3:** Hybrid techniques in analyzing MR images.

Technique	Ref.	Target	Result
Wavelet transform (WT), Genetic algorithm (GA) and supervised learning methods (SVM).	[76,77]	Classification of brain tissues in MRI images	This technique is accurate, Easy to operate, Non-invasive and inexpensive.
K-means, Sobel edge detection and morphological operations.	[78]	Segmentation of Brain Lesions in MRI and CT Scan Images	Achieves a high accuracy 94% in compared with manual delineation performed.
Support vector machine (SVM) and Fuzzy c-means (FCM).	[79,80]	Detection of Brain Tumor in MRI Images	Provide accurate and more effective result for classification of brain MRI images in minimal execution time.
K-Means, Nonsubsampled contourlet transform (NSCT) and SVM.	[81]	MRI Brain Tumor Images Classification	Higher classification accuracy.
K-Means, Gray Level Co-occurrence Matrix (GLCM), Berkeley Wavelet Transform (BWT), Principal Component Analysis (PCA) and Kernel Support Vector Machine (KSVM).	[82]	Detection and classification of MRI images	proposed method can be used for clinical purpose for screening and then diagnosed by the radiologists with high performance and accuracy.
Fuzzy Clustering, Gabor feature extraction and ANN.	[83]	Detection and Classification for Brain tumor	The classifier’s output helps the radiologist to make the decisions without any hesitation and achieved classification accuracy of 92.5%.

**Table 4:** Comparison of CNN modalities.

Scheme	Dataset	Ref.	Ways of Training and Testing	Achievement
Rely on CNN	BRATS 2015 and ISLES 2015	[7]	Dual pathway	An efficient solution processing for multi-scale processing for large image context using parallel convolutional pathways.
	BRATS 2017 and BRATS 2015	[68]	Dual-force	For learning high-quality multi-level features used a dual-force training strategy
	BRATS 2013 and BRATS 2015	[65]	Patch-based	Used 3 × 3 kernels to permit deeper architectures for CNN-based segmentation method for brain MRI images.
Rely on DCNN	ImageNet LSVRC-2010	[96]	Patch-based	Gained top-1 and top-5 error rates of 37.5% and 17.0%
	ISBI 2012& 2015	[95]	End-to-end	Enabled precise localization.
	BRATS 2013	[71]	T1, T1c, T2 and FLAIR images	3D segmentation problem is converted into triplanar 2D CNNs.
	BRATS 2013	[4]	T1, T1c, T2 and FLAIR images	Novel CNN architecture which improved accuracy and speed as presented in MICCAI 2013.
Rely on FCN	BRATS 2013 & 2016	[17]	T1, T1c, T2 and FLAIR images	Integration of FCN and Conditional Random Fields for brain tumor segmentation.
	BRATS 2013	[97]	End-to-end	Improve brain tumor segmentation performance by a symmetry-driven FCN
	ISBR and ABIDE (17 different sites)	[5]	End-to-end	Used 3D convolutional filters and FCN for an automatic segmentation of subcortical brain regions.

**Table 5:** Comparison of different architecture of CNN.

Ref.	Architectures	Layers	Advantages	Disadvantages
[102]	LeNet-5	7 layers	Ability to process higher resolution images need larger firmer layers.	Overfitting in some cases and no built-in mechanism to avoid this
[103]	AlexNet	8 layers 60 M parameters	A very rapid downsampling of the intermediate representations through convolutions and max-pooling layers.	The use of large convolution filters (5 × 5) is not encouraged shortly after that, Is not deep enough rather than another techniques.
[104]	ZFNet	8 layers	Improved image classification rate error in compared with Alexnet, winner of ILSVRC2012	Feature maps are not divided across two different GPU, Thus connections between layers are dense.
[103]	GoogleNet	22 layers 4–5 M parameters	Winner of ILSVRC2014, Decreased the number of parameters from 60 million (AlexNet) to 4 million so network can have a large width and depth.	Consists of a hierarchy of complex inception modules/blocks that consist of operations over different scales in each of the modules.
[105]	VGGNet	Between 11 to 19 layers the best one is 16 layers 138 M parameters	At present it is the most prefer election for extracting features from images.	Consists of 138 million parameters, which can be a bit challenging to handle.
[105]	ResNet	152 layers	Network learns difference to an identity mapping (residual), Faster convergence if identity is closer to the optimum.	Lower complexity than VGGNet, Overfitting would increase test but decrease training error.

**Table 6:** Architectures of CNN and their targets.

Architectures	Examples	Target	Accuracy
LeNet-5	[106]	Detection of brain cancer by tensorflow	99%
	[38]	classify Alzheimer’s brain	96.85%
Alex Net	[107]	Lung nodules in chest X-ray	64.86%
	[108]	Diagnosis of Thyroid Ultrasound Image	90.8%
	[109]	Classification of skin lesion	96.86%
VGGNet-16	[110]	Brain tumor classification	84%
	[111]	Diagnosis of Prostate Cancer	95%
Google Net	[112]	Thyroid Nodule Classification in Ultrasound Images	98.29%
	[107]	Lung nodules in chest X-ray	68.92%
ResNet	[113]	Brain tumor classification	89.93%
	[114]	Pancreatic tumor classification	91%
ZefNet	[115]	The trends and challenges for future edge reconfigurable platforms of deep learning.	

**Table 7:** CNN methods in medical domain.

Ref.	Features	Methods	Testing Sample	Achievement	Accuracy
[112]	Type, size, shape, tumor features	DCNN and googleNet	Thyroid nodules	Improving the performance of fine-tuning and augmenting the image samples.	98.29%
[91]	Size, tumor features, doughnut-shaped lesion	FCN, VGG-16, U-Net	Colorectal tumors	Can remodel the current, time-consuming and non-reproducible manual segmentation method.	-
[114]	Type, size	ResNet18, ResNet34, ResNet52 and Inception-ResNet	Pancreatic Tumors	ResNet18 with the proposed weighted loss function method achieves the best results to classify tumors.	91%
[117]	Type, size, shape	CAD system using a multi-view convolutional network	Pulmonary Nodule	Boosts the detection sensitivity from 85.7% to 93.3%.	-
[38]	Shape, scale	CNN and LeNet-5	Alzheimer’s disease classification	Possible to generalize this method to predict different stages of Alzheimer’s disease for different age groups.	96.85%
[109]	Type, color image lesions	transfer learning and Alex-net	skin lesions classification	Higher performance than existing methods.	96.86%
[111]	Image lesion, type	VGGNet and patch-based DCNN	Prostate cancer	Enhanced prediction	95%
[119]	Textures	AlexNet	Breast cancer	Showed that accuracy obtained by CNN on BreaKHis dataset was improved.	-

**Table 8:** Existing challenges and potential solutions.

Existing Challenges	Example	Ideas as Potential Solution
Often classification or segmentation in medical imaging is introduced as a binary task, normal versus abnormal, object versus background.	In some rare situations, normal tissues and categories can find benign categories.	By presenting accurate annotations of all possible subclasses, we can convert the deep learning system into a multi-class system [39].
Depending on the task performed in medical imaging, images for the unusual class might be challenging to find.	Most cancerous lesions do not cause death; in mammograms, a suspicious lesion is usually not cancerous.	Conducted a thorough evaluation of data augmentation strategies for lesion segmentation [65].
In a deep learning network, balancing between the number of imaging features with the number of clinical features is a challenge.	Physicians, for an accurate diagnosis, usually need to use descriptive information.	Connect the whole image to the deep network and use different types of evaluation to guide learning [39].
In CAD, the biggest challenges are the diversity in shape and intensity of tumors or lesions as well as the existence of differences in the imaging protocol in the same imaging modality.	Use of simpler machine learning appeals in Rician noise, non-isotropic resolution, and bias field effects. Automatic handling is not usable in MRI.	For classification of hand-designed features in a through, separate step, conventional machine learning approaches are trained [138].
Deep learning does not leave a search trail to clarify its decisions, so it is considered a black box.	To specify an exact feature, such as an edge, circle, or class activation maps (CAMs), that localizes the important regions in an input used for the prediction.	Feature visualization is a feature which is identified in the feature maps. Attribution is a part of the input responsible for the corresponding prediction [84].

## Data Availability

The study did not report any data.
